# Aneurism of the Abdominal Aorta

**Published:** 1845-03

**Authors:** 


*Aneurism of the Abdominal Aorta.*—Dr. Branson, at a meeting of the Sheffield Medical Society, exhibited a specimen of aneurism of the abdominal aorta, taken from an infirmary patient, aged thirty three, a table-blade forger, of intemperate habits, who died suddenly on the 23d of October. The aneurismal tumour originated nearly opposite the point where the cæliac artery is given off; the sac was of considerable size, and embraced four of the vertebræ, the bodies of which were deeply excavated. The sac itself was ruptured in two places, and more than a quart of coagulated blood was found between the peritoneum and the muscles; the left kidney was buried in the clot. The man continued at his employment till within five days of his death, and complained only of slight symptoms of nephralgia. The case was instructive, as showing how great may be the mischief, and how comparatively trifling the symptoms. The anterior walls of the thorax of this patient, presented a curious congenital malformation; the sternal extremity of the third rib on the left side, was wanting for the space of four inches, and the fourth rib was completely separated from its cartilage; the vacant space was occupied by a strong tendinous expansion.—Through the centre of the sternum also there was an opening, the size of a sixpence. The man had never received any local injury.—*Ibid.*

